# miR-29a inhibits human retinoblastoma progression by targeting STAT3

**DOI:** 10.3892/or.2021.8126

**Published:** 2021-06-28

**Authors:** Shu Liu, Xiaomeng Zhang, Chunmei Hu, Yingxue Wang, Chunling Xu

Oncol Rep 39: 739-746, 2018; DOI: 10.3892/or.2017.6144

Following the publication of this article, an interested reader drew to the authors attention that some western blotting data bands had apparently been duplicated in Fig. 4D. The authors have re-examined their original data, and realized that this figure was assembled incorrectly.

The corrected version of Fig. 4 is shown below. The authors sincerely apologize for the errors that were introduced during the preparation of this figure, and thank the Editor for allowing them the opportunity to publish a Corrigendum. Furthermore, they regret any inconvenience caused to the readership.

## Figures and Tables

**Figure 4.**
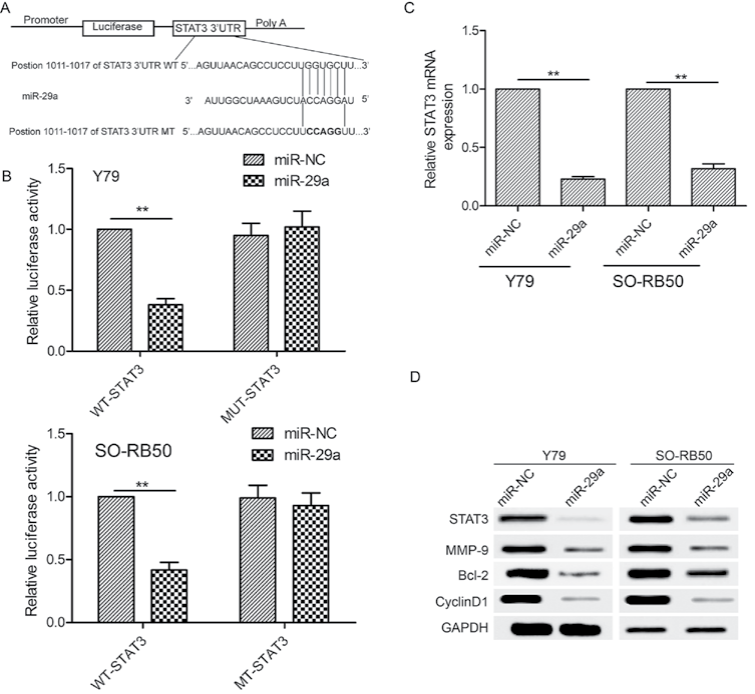
STAT3 is a potential direct target of miR-29a in RB. (A) The predicted binding sites and mutation sites for miR-29a in the 3′-UTR of STAT3 are shown. (B) miR-29a overexpression significantly decreased the luciferase activity of the WT-STAT3-3’-UTR but not that of the mutant STAT3-3′-UTR in Y79 and SO-RB50 cells. (C) Overexpression of miR-29a reduced the STAT3 mRNA expression levels in Y79 and SO-RB50 cells. GAPDH was used as an internal control. (D) Overexpression of miR-29a reduced the STAT3, cyclin D1, Bcl-2 and MMP-9 protein levels in Y79 and SO-RB50 cells. GAPDH was used as an internal control. **P<0.01..

